# Optic Nerve Glioblastoma with Optic Chiasm Involvement: A Case Report and a Brief Literature Review

**DOI:** 10.3390/medicina60101687

**Published:** 2024-10-14

**Authors:** Artis Brokāns, Jūlija Dolgopolova, Agnis Saulītis, Uldis Spulle, Kristaps Rancāns, Dairis Meiers, Sigita Hasnere, Arturs Balodis

**Affiliations:** 1 Department of Radiology, Riga Stradins University, 16 Dzirciema Street, LV-1007 Riga, Latvia; dr.artis.brokans@gmail.com; 2 Institute of Diagnostic Radiology, Pauls Stradins Clinical University Hospital, 13 Pilsonu Street, LV-1002 Riga, Latvia; 3 Department of Neurosurgery, Pauls Stradins Clinical University Hospital, 13 Pilsonu Street, LV-1002 Riga, Latvia; julija.dolgopolova@stradini.lv (J.D.); agnis.saulitis@stradini.lv (A.S.); kristaps.rancans@stradini.lv (K.R.); 4 Department of Oral and Maxillofacial Surgery, RSU Institute of Stomatology, LV-1007 Riga, Latvia; uldis.spulle@stradini.lv; 5 Department of Ophthalmology, Pauls Stradins Clinical University Hospital, 13 Pilsonu Street, LV-1002 Riga, Latvia; dairis.meiers@stradini.lv; 6 Department of Oncology, Pauls Stradins Clinical University Hospital, 13 Pilsonu Street, LV-1002 Riga, Latvia; sigita.hasnere@gmail.com

**Keywords:** optic nerve glioblastoma, radiology, neurosurgery, ophthalmology

## Abstract

*Background*: optic nerve glioblastoma is an uncommon pathology. The optic chiasm, optic tract, or optic nerves are possible places from which the tumor can originate. Most of the neuroimaging findings are nonspecific. To confirm the diagnosis, a biopsy is required. A delay to the treatment plan for optic nerve glioblastoma results in poor patient survival rates. *Case report*: a 68-year-old woman with an uncomplicated medical history presented with exophthalmos, deteriorating eyesight, and partial loss of vision. Using radiological data together with postoperative histopathological and histochemical analysis, optic nerve glioblastoma, IDH-wildtype, with optic chiasm involvement was diagnosed. *Conclusion*: optic nerve glioblastoma is a rare and aggressive form of cancer that affects the optic nerve, leading to significant vision impairment and potentially life-threatening complications. Treatment options are restricted and difficult because of the location and nature of the condition; surgery, radiation therapy, and chemotherapy are frequently needed as part of a multidisciplinary approach.

## 1. Background

About 1.2 million axons are found within the optic nerve, which are responsible for vision [1]. Adult-onset optic nerve glioma is an uncommon condition, representing only about 0.6–1.2% of all brain tumors [2]. Most of the gliomas are benign and approximately 90% of them occur in children, in the first decade of life, while the remaining occur in adults and are usually malignant [3].

Malignant optic nerve gliomas were initially identified and outlined in 1973 by Hoyt et al. Most patients tend to be middle-aged males who begin with a steadily declining vision that eventually results in blindness in a matter of weeks or months [4]. The optic chiasm, optic tract, or optic nerves are possible places from which the tumor can originate. Despite the findings being nonspecific, magnetic resonance imaging (MRI) of the brain is the primary diagnostic tool for optic nerve lesions. To confirm the diagnosis, a biopsy is required.

In this paper we present a patient diagnosed with optic nerve glioblastoma, a neuronal tumor with glial cell origin, that has been assigned to WHO Classification Grade 4 [5]. This paper also provides a brief overview of the clinical symptoms, ophthalmological findings, neuroradiological results, surgery, and pathology report and compares them to instances documented in the literature up to this point.

## 2. Case Report

A 68-year-old woman presented to the hospital with painless outward protrusion of the left eye which first manifested six months earlier, accompanied by deteriorating eyesight, limited ability to look upward, and partial loss of vision in the same eye. The patient had no other past illnesses or family history of the same. Additionally, the patient reported that the first indications of deteriorating vision began in the year 1978. The patient was examined by the ophthalmologist and scheduled for a noncontrast enhanced computed tomography (CT) scan of the patient’s orbits.

An extensive and thorough ocular examination was performed on both eyes, encompassing ocular ultrasonography, optical coherence tomography (OCT), slit lamp and torch light examinations, and visual acuity assessments. The tests revealed exophthalmos, ptosis, optic nerve atrophy, senile macular degeneration, and corpus vitreum destruction of the left eye. A visual acuity test showed +2.75 DSph (diopter sphere) in both eyes. There was also acknowledgement of limited left eye movement.

**Figure 1:**
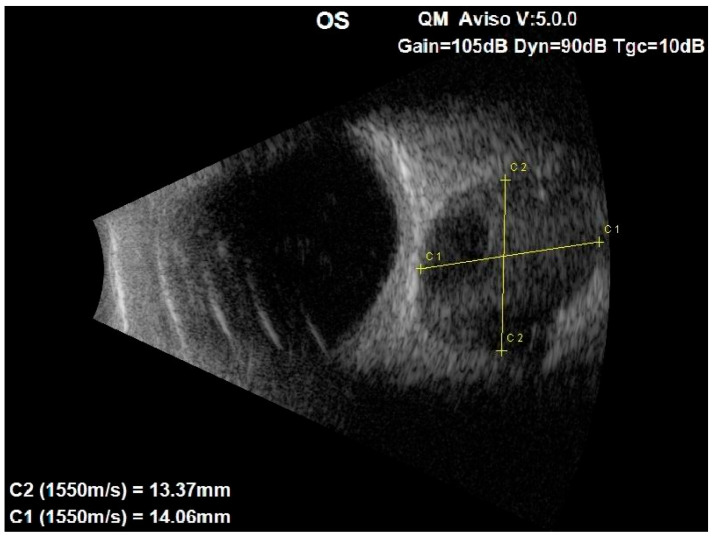
Ocular ultrasound of the left eye showing a centrally located retrobulbar tissue echo signal mass, measuring about 1.3 cm × 1.4 cm.

**Figure 2:**
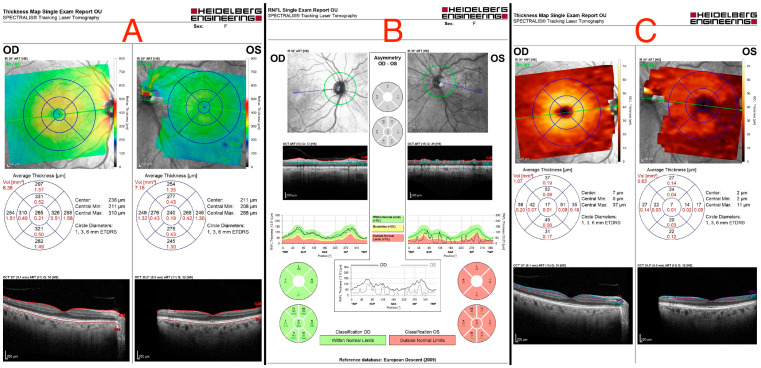
(**A**) Optical coherence tomography of both eyes, showing a flattened thinner retina around the macular area in the left eye. (**B**) Retinal nerve fiber layer (RNFL) analysis of both eyes, revealing a reduced retinal nerve fiber layer in all left eye’s quadrants. (**C**) Ganglion cell layer (GCL) measurement showing asymmetry in both eyes, indicating ganglion cell damage in the left eye.

An ocular ultrasound showed a tissue type echo signal mass, measuring about 1.3 cm × 1.4 cm, behind the left eye (Figure 1). Optical coherence tomography (OCT), retinal nerve fiber layer (RNFL) analysis, and ganglion cell layer (GCL) measurement were all performed (Figure 2). These revealed a flattened thinner retina around the macular area in the left eye and a reduced retinal nerve fiber layer in all left eye’s quadrants, reflecting the loss of ganglion cell axons. By assessing the ganglion cell layer thickness in the left eye’s macular area, ganglion cell damage was detected.

**Figure 3:**
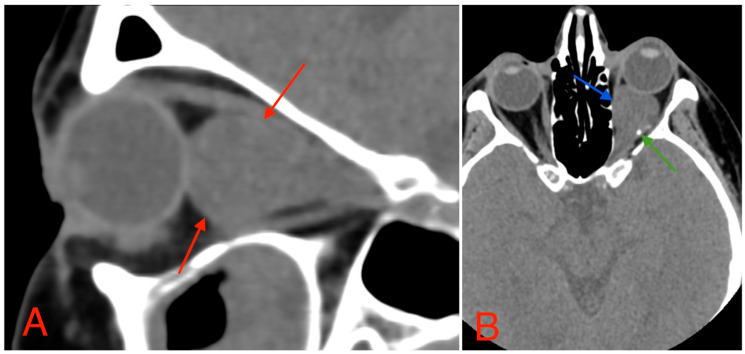
(**A**) CT scan of orbits, sagittal plane, 2 mm slice, showing a homogeneous, well-marginated optical nerve tumor with a mass effect and a compression of the rectus muscles of the eye (red arrows). (**B**) CT scan of orbits, axial plane, 1.25 mm slice, showing an intraconal lesion with intracranial extension, mass effect (blue arrow), and small calcification (green arrow). A mild exophthalmos can also be noted.

**Figure 4:**
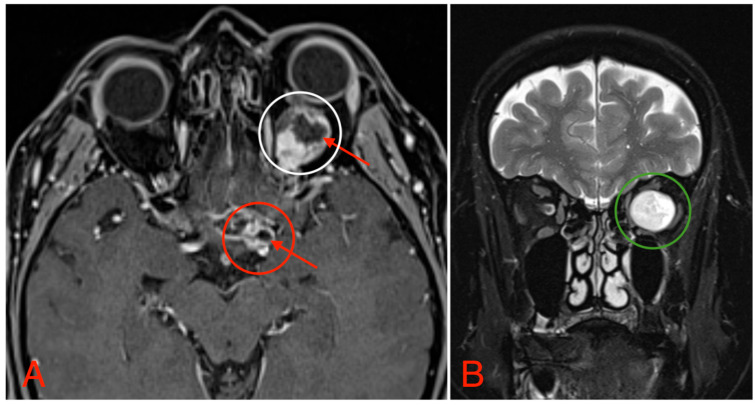
(**A**) MRI image, T1 VIBE fat-saturated post-gadolinium image sequence, axial plane, demonstrating an intraconal tumor which most likely originated from the optic nerve with intraconal and intracranial involvement, cystic changes (red arrows), and contrast enhancement (white circle). The intracranial compartment involves the optic chiasm with a mass effect on the surrounding structures and large blood vessels (red circle). (**B**) MRI image, T2 TSE Dixon sequence, coronal plane, showing both solid and cystic parts of the optic nerve tumor with a retrobulbar segment mass effect and extraocular muscle suppression (green circle).

The CT scan revealed a pathological mass in the intraconal space, ~1.5 cm × 2.5 cm × 1.5 cm, which occupied most of the orbit. Noticeable thinning and depression of the rectus muscles of the eye were also noted (Figure 3). A cranial contrast enhanced MRI was conducted the following month, revealing an entirely altered optic nerve affecting the retrobulbar, prechiasmal, and optic chiasm level. A pathological, smoothly contoured mass with cystic changes was described and measured about 5.2 cm × 2.0 cm × 1.9 cm. Greater peripheral contrast accumulation in the pathological tissue extended along the optic chiasm and relatively close to the frontal lobe’s straight gyrus. The pathological mass also extended relatively close to the A1 segment of the anterior cerebral artery and stretched in the direction of the pituitary gland. A lateral depression of the extraocular muscles was also noted (Figure 4). The contrast-enhancing component of the tumor on diffusion-weighted imaging (DWI) sequence showed a restricted diffusion at an apparent diffusion coefficient (ADC) value of 1500 × 10^−6^ mm^2^/s, with a b value of 1000 s/mm^2^. A high T2 signal intensity was also noted.

A probable diagnosis of optic nerve glioma was acknowledged in the MRI report’s conclusion. Surgical resection was proposed to the patient for extirpation of the tumor via pterional craniotomy. The patient agreed to the surgery.

### 2.1. Surgery and Pathology

After the patient received anesthesia and before positioning, a lumbar drainage was inserted to reduce the volume of the cerebrospinal fluid for better visualization during the operation.

**Figure 5:**
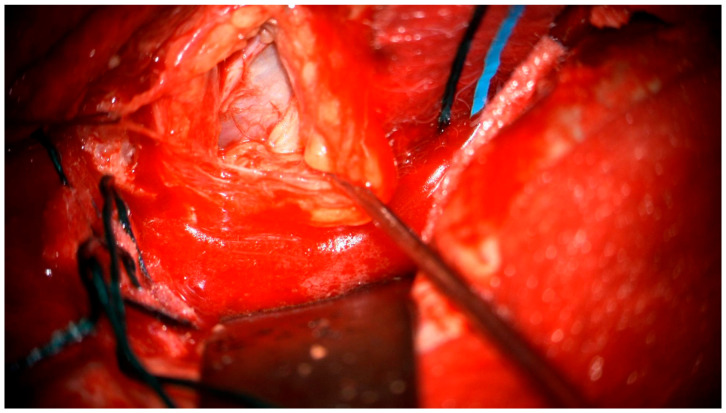
Surgical microscope view of the surgical site.

After the lumbar drainage was inserted, the patient was positioned in a supine position, with the head fixated in a Mayfield clamp and turned to the right. Neuronavigation based on previous MRI was used to mark the incision. A left-sided pterional craniotomy was performed to access the eye socket; a resection of part of the superior orbital wall was performed. With a surgical microscope, the intraorbital dura mater was opened. The pathological tissues appeared in a light gray color, tissues were loose, and the tumor was fragmented and aspirated with the help of a CUSA (cavitronic ultrasonic surgical aspirator) (Figure 5). After closure of the dura mater, the orbital wall was reconstrued with an individually mended standard titan orbital wall implant, which was fixated with three mini screws. Under the microscope, pathological tissues in the medial brain fossa were visualized and resected subtotally.

**Figure 6:**
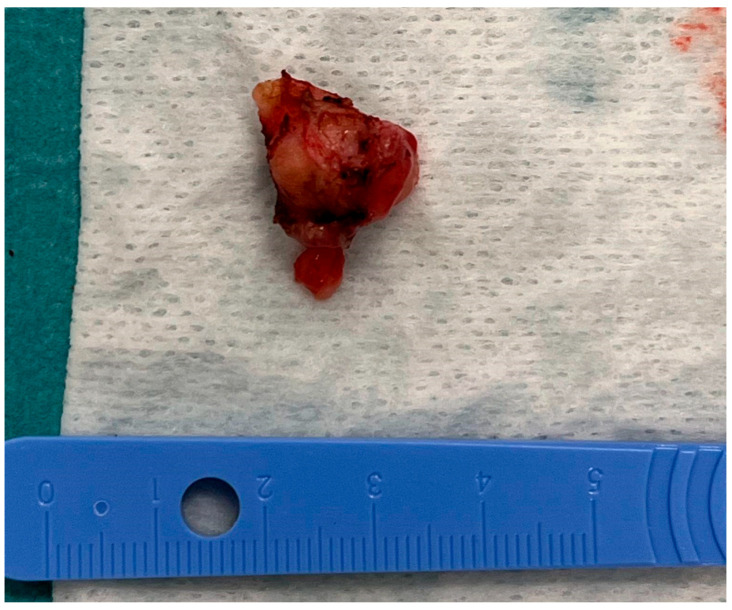
The tumorous tissue extracted during the surgery.

The tumorous tissue (1.5 cm × 0.5 cm × 0.3 cm) was extracted during the surgery and delivered to the pathology laboratory (Figure 6).

**Figure 7:**
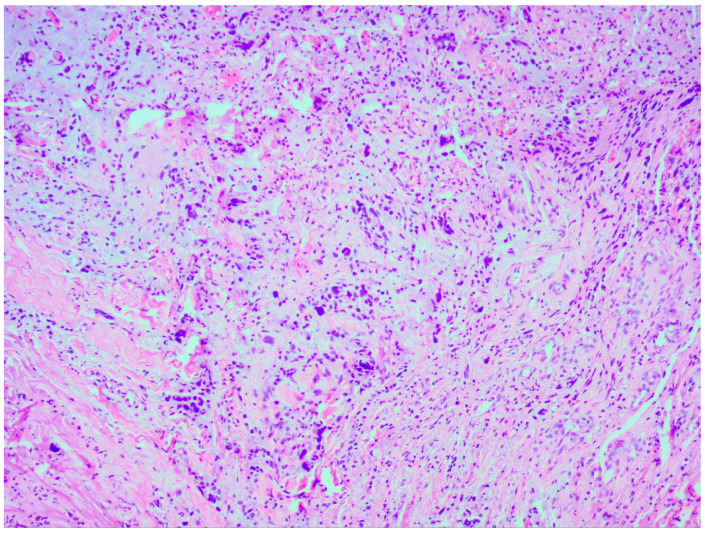
Microscopic image of histopathological analysis, showing spherical cells with pronounced nuclear polymorphism, multinucleated tumor cells, and an area of vascular proliferation.

Thorough histopathological analyses highlighted the moderate formation of cellular material, consisting of spherical cells with pronounced nuclear polymorphism, multinucleated tumor cells, and an area of vascular proliferation (Figure 7). Multiple mitotic figures were visualized within the tissue. A histochemical analysis of the tumor tissue revealed the presence of positive glial fibrillary acidic protein (GFAP). Alpha thalassemia intellectual disability syndrome X-linked (ATRX) and isocitrate dehydrogenase 1 (IDH 1 R132H) were both negative. Antigen Kiel 67 protein (KI-67) was positive, but not elevated, around 20–25%.

Histopathological and immunohistochemical examination findings revealed a diagnosis of glioblastoma, IDH-wildtype, WHO Classification Grade 4.

### 2.2. Outcome and Follow-Up

Complications were not observed during the early postoperative phase. After the operation, full blindness of the left eye, oculomotor nerve paresis, left sided ptosis, and no major change in the exophthalmos were noted. The patient was assigned to a physiotherapist.

**Figure 8:**
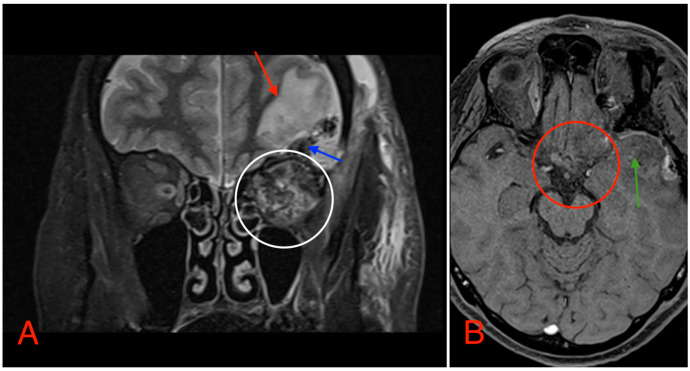
(**A**) MRI image, T2 TSE STRI sequence, of the aftermath of the left-sided pterional craniotomy, showing postsurgical edema (red arrow) with a small hemorrhagic component (blue arrow). Postsurgical changes in the intraconal space can also be noted, showing an edema and a hemorrhage (white circle). (**B**) MRI image, T1 vibe post-gadolinium image sequence, showing postsurgical changes in the region of the optic chiasm with partial (subtotal) tumor resection (red circle). Local brain edema with hemorrhagic component (green arrow) can also be noted.

Postoperative head MRI showed partial resection of the left optic nerve. Residual tumor tissue and edema were noted in the left retrobulbar space, extending into the left prechiasmal space and into the left side of the optic chiasm (Figure 8).

Based on the morphological and immunohistochemical examination data, as well as the radiological postoperative data, a diagnosis of glioblastoma with optic chiasm involvement was determined.

The patient was scheduled for radiation and temozolomide chemotherapy. As we are writing this paper, the patient has received a little over half of the radiation dosage that has been planned. A total of 60 Gy (gray) units in 30 fractions over a period of six weeks were scheduled. A total of 120 mg of temozolomide combined with 8 mg ondansetron were also prescribed for daily administration. As of yet, the patient has not noted any discomfort and the treatment plan is proceeding as scheduled.

## 3. Discussion

A review by Wabbels et al. (2004) reported around 44 cases of optic nerve glioblastoma, highlighting its rarity and the challenges associated with its diagnosis and management [6]. Another more recent review by Traber et al. (2015) reported an additional 21 cases, bringing the total number of cases to around 70 [7]. Years have passed since those papers were published, many more case reports have been published around this topic since then, slightly increasing the total number of reports; however, the overall prevalence of the disease remains extremely low, often leading to these cases being discussed in case reports or small series rather than in large-scale studies.

Even though neuroimaging features are nonspecific in the early to mid-staged diagnosis of the disease, the preferred method of imaging is MRI, usually performed using an intravenous contrast enhancement [8]. Still, a brain computed tomography is typically performed on the patient before the MRI. As most of the medical literature on the disease states, the two most commonly reported MRI findings include enhancement of the tumor in the postcontrast T1 sequence and high T2 signal of the tumor [9]. Both findings were observed in our presented case.

Differential diagnosis of optic nerve glioblastoma can include optic nerve schwannomas, optic nerve sheath meningiomas, and optic nerve lymphomas. Just as the optic nerve glioblastoma, optic nerve schwannomas exhibits a nonspecific clinical presentation that most commonly include abnormalities in visual acuity, orbital discomfort, and exophthalmos. Schwannomas most often show a low signal intensity on T1 weighted images and a high signal intensity in T2 weighted images, accompanied by homogeneous contrast enhancement in the MRI imaging modality [10]. Both T1 and T2 weighted images show optic nerve sheath meningiomas as isointense to gray matter with strong postcontrast enhancement [11]. In T1 weighted images, optic nerve lymphomas often seem isointense to hypointense, while, in T2 weighted images, they appear to be hypointense [12]. The lack of distinctive imaging features often necessitates further investigation to rule out other diseases.

The aggressive nature of the optic nerve glioblastoma and its sensitive placement make treatment difficult. Although the prognosis for optic nerve glioblastoma remains poor, standard treatment options often include a combination of radiation therapy, chemotherapy, and surgery.

Since complete resection is typically not feasible due to the proximity of the tumor to the brain and to visual structures, the surgical treatment strategy for optic nerve glioblastoma focuses on tumor debulking to minimize symptoms and on biopsy for diagnosis. In most cases, especially when the tumor extends into the optic chiasm as in our patient, the goal of surgery is to remove as much of the tumor as possible without seriously impairing neurological functioning or vision. Surgical intervention is carefully weighed against the possible benefits of debulking due to the significant risk of postoperative complications, including loss of vision. While surgical precision has increased due to technological advancements like neuronavigation and intraoperative imaging, the prognosis remains poor [13].

In this case, a surgical microscope and neuronavigation were used. Use of these modalities greatly improves safety and precision. Surgical microscopes allow surgeons to perform more precise resections, particularly in delicate locations like the optic nerve, by providing enlarged, high-resolution pictures of the tumor and the surrounding tissues. Through the integration of preoperative imaging data (such as MRI or CT scans), neuronavigation functions as a real-time GPS, guiding the surgeon. This technology is essential for reducing functional impairments and enhancing the overall surgical outcomes, since it guarantees precise tumor targeting while minimizing injury to nearby healthy tissues.

To slow tumor growth and increase survival time, radiation therapy and temozolomide chemotherapy are the hallmarks of treatment for optic nerve glioblastoma. Radiation therapy is used to target the remaining tumor cells after surgery. Typically, 60 Gy is administered in 30 fractions over a period of six weeks. This is an efficient way to stop the growth of localized tumors, but its effectiveness is constrained by the possibility of damaging nearby healthy tissues, such as the brain and the remaining optic nerve. Since temozolomide has been shown to be effective in increasing the survival rates of glioblastoma patients, it is now used in conjunction with radiation therapy and is considered the standard of care. To further lower the chance of recurrence, six rounds of adjuvant temozolomide are usually administered after this concurrent therapy [14,15].

Glioblastomas typically relapse within the high dose radiation field, which is known as the peritumoral brain zone (PTZ), demanding further treatment after the initial course of treatment. PTZ is characterized by the presence of tumor and stromal cells that encourage the growth and invasion of GBM in a particular parenchymal region. There is no established protocol for treating patients with recurrent glioblastoma, and their median survival is less than a year. Currently, reirradiation is a practical local strategy that may be used in place of or in addition to surgery [16].

Advancements in patient outcomes, quality of life, and treatment methods personalization are the main goals of innovations in the management of patients with glioblastoma in any region. AI (artificial intelligence) is being used to evaluate imaging data to distinguish between treatment effects and tumor growth, enhancing decision-making and allowing for earlier modifications to treatment plans [17,18].

Based on the unique genetics of each patient’s tumor, targeted drugs are revolutionizing patient care. Next-generation sequencing for tumor profiling makes it possible to identify alterations in the genes which may influence therapeutic choices. With the aid of personalized medicine, treatments like immunotherapy or targeted inhibitors can be specifically tailored to a tumor’s unique genetic modifications [19].

## 4. Conclusions

The optic nerve is a region where glioblastoma has rarely been documented in the literature. This case report of optic nerve glioblastoma adds new information about this uncommon manifestation of the disease. It sheds light on the diagnostic difficulties caused by its unusual presentation, which can resemble other illnesses of the optic nerve, therefore contributing to the advancement of differential diagnosis. The example also highlights the significance of neuroradiology and histopathological analysis for any progressively growing lesion in the optic nerve. Lack of distinctive imaging findings and of a clear clinical presentation makes early identification challenging. A delay to the treatment plan for optic nerve glioblastoma results in poor patient survival rates. The little information currently available on the prognosis and treatment options for optic nerve glioblastomas is improved by this publication, which may help direct future clinical initiatives.

## Data Availability

The data presented in this study are available upon request from the corresponding author.
